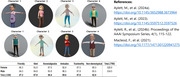# Co‐creation Design of Virtual Agents for Reminiscence Therapy in Dementia Care: the case of AMPER ‐ Agent‐based Memory Prosthesis to Encourage Reminiscence

**DOI:** 10.1002/alz70863_110511

**Published:** 2025-12-23

**Authors:** Mario A A Parra, Meiyii Lim, Matthew A Jamieson, Bruce W. Wilson, Lynsey A Stewart, Ruth Aylett, Matthew Aylett

**Affiliations:** ^1^ University of Strathcylde, Glasgow, Glasgow UK; ^2^ Heriot‐Watt University, Edinburgh UK; ^3^ University of Strathclyde, Glasgow UK; ^4^ Heriot‐Watt University, EDINBURGH UK

## Abstract

**Background:**

Reminiscence therapy can improve emotional and cognitive engagement in individuals with dementia, thus restoring their sense of self‐worth, self‐esteem, and quality of life (Macleod et al., 2021). Embodied Conversational Agents (ECAs) offer a novel way to facilitate this therapy by providing consistent, personalised, and engaging interactions (Aylett et al., 2024 a&b). Reminiscence technologies should emerge from co‐design methodologies, which emphasise collaboration with users and caregivers to create theory‐driven and user‐led systems that are intuitive, engaging, and impactful. Here, we report on the co‐creation design approach of AMPER.

**Methods:**

The co‐design process incorporated user‐centred design (UCD) and participatory design (PD) methodologies. Workshops and interviews were conducted with caregivers, clinicians, and other stakeholders to define key design requirements. Prototypes were developed iteratively, integrating feedback to refine visual design, interaction flow, and narrative content. ECAs prototypes were tested for their suitability, using scales to measure the reliability of traits (trustworthiness, friendliness, kindness, knowledgeability, amicability, and non‐stereotypical appearance) and engagement. Natural language processing and multimedia prompts were used to enhance personalisation and contextual relevance.

**Results:**

Out of 17 ECAs, 8 were shortlisted by 6 raters. The resulting female (*n* = 4) and male (*n* = 4) ECAs attracted high ratings on trustworthiness (87.6/130), friendliness (87.3/130), kindness (87.9/130), knowledgeability (86.4/130), amicability (89.6/130), and non‐stereotypical (82.9/130) from cognitively unimpaired older adults (*n* = 26 for female ECA and *n* = 28 for male ECA, age range 59‐87). Trustworthiness showed correlations (Spearman's rho 0.3−0.4) with the other traits but non‐stereotypical. Intra‐class Correlation Coefficient (ICC) was 0.88 (*p* <.001, 95% CI=0.73‐0.97), suggesting high inter‐rater agreement. Participants reported increased emotional engagement, citing the value of personalised ECAs, narratives, and multimedia integration.

**Conclusions:**

The AMPER co‐creation design approach proved effective in creating tailored ECAs for reminiscence therapy (Aylett et al., 2024b). We provide guidelines for future evaluation of intelligent ECAS in a co‐design context (Aylett et al., 2024a). This research contributes to the growing field of trustworthy autonomous systems for healthcare applications, emphasising interdisciplinarity, user trust, and engagement as critical factors for success.